# Effectiveness of SMILE Combined with Micro-Monovision in Presbyopic Patients: A Pilot Study

**DOI:** 10.3390/life13030838

**Published:** 2023-03-20

**Authors:** Joaquín Fernández, Federico Alonso-Aliste, Noemí Burguera, Julia Hernández-Lucena, Jonatan Amián-Cordero, Manuel Rodríguez-Vallejo

**Affiliations:** 1 Qvision, Ophthalmology Department, VITHAS Almería, 04120 Almería, Spain; 2 Tecnolaser Clinic Vision, Ophthalmology Department, 41018 Sevilla, Spain

**Keywords:** presbyopia, micro-monovision, SMILE, efficacy, binocular summation

## Abstract

Binocular summation along all defocus range after a micro-monovision procedure has scarcely been studied. The aim of this pilot study was to evaluate the efficacy of SMILE combined with different levels of micro-monovision in presbyopic patients and to assess the binocular summation effect on contrast sensitivity defocus curves (CSDC) at the 6-month follow-up. Efficacy was assessed on the basis of visual acuity (VA) and stereopsis at far, intermediate, and near distances. Patient-reported outcomes (PROs) and binocular CSDC were also evaluated. Six patients completed the study with a programmed median anisometropia of 0.81 Diopter. The median binocular uncorrected VA was better than 0 logMAR at the three evaluated distances, and stereopsis was not impaired in any patient, achieving a median of ≤119 arcsec at any distance. CSDC increased binocularly after surgery, significantly in the range of −2 to −3 D (*p* < 0.05). No clinically relevant changes were observed in PROs compared with the preoperative period, and all patients achieved spectacle independence at intermediate/near distance and were likely or very likely to undergo the same surgery. In conclusion, micro-monovision with SMILE could be an effective procedure, with results that might be comparable to other laser correction techniques specifically designed for presbyopia correction.

## 1. Introduction

Presbyopia is a global age-related vision disorder characterized by a progressive inability to focus on nearby objects. It is estimated that about 209 million people suffer from presbyopia in Europe (44% of the population) and it is expected to affect half of the European population by 2030 [1]. Monovision is one of the available clinical techniques for correcting the visual limitations caused by presbyopia. This procedure consists of correcting the residual refractive error that causes loss of distance vision in one eye, while the other eye corrects the residual refractive defect in near vision [2]. Then, if a lower degree of anisometropia is induced (≤1.5 D), this protocol is called micro-monovision [3]. In the particular case of coexisting myopia and presbyopia, the total correction of myopia is usually applied in one eye, while the other eye is not fully corrected, leaving a residual myopia that ranges from −0.50 to −1.50 D depending on the patient’s tolerance to the residual anisometropia. Complete correction of myopia in one eye and undercorrection of myopia in the other eye allows the patient to achieve better near vision than full correction of myopia in both eyes. In a monovision-adapted patient, this is because each eye dominates the other eye, according to the visual task. For example, when a patient performs tasks that require good distance vision, such as driving, the fully corrected eye dominates the undercorrected eye. On the other hand, when performing intermediate- or near-vision tasks, such as computer vision or reading, the eye undercorrected at a far distance dominates over the fully corrected eye since undercorrection favors this eye to have a higher visual acuity for the development of intermediate/near tasks. In conclusion, micro-monovision involves adapting the patient’s refraction to achieve the best possible vision at all distances with the alternating use of both eyes.

Small incision lenticule extraction (SMILE) is a laser refractive surgery (LRS) technique that allows the correction of myopia and astigmatism. SMILE involves creating a lenticule inside the cornea (stroma) using a femtosecond laser, which is removed by a micro-incision of approximately 2 mm. Safety, efficacy, and predictability of SMILE for the correction of myopia and astigmatism has been widely reported, demonstrating equivalent results to laser-assisted in situ keratomileusis (LASIK), but with some advantages such as less induction of higher order aberrations and less affectation of ocular dryness [4,5].

Although micro-monovision studies have been conducted with older LRS techniques such as LASIK or photorefractive keratectomy (PRK) [2], few studies have been conducted with the combination of SMILE and micro-monovision, mainly because it is a more recent technique [6,7,8]. In addition, although the usual clinical practice procedure involves the correction of distance vision in the dominant eye and near vision in the non-dominant eye [6,7,8], recent laboratory research suggests that the vision of a patient undergoing a monovision procedure may vary according to different patterns of accommodative response [9], as presbyopic patients in their 40s and 50s still retain some accommodative capabilities. Thus, depending on the accommodative response, the patient may achieve a different range of clear vision. This pilot study aimed to evaluate the efficacy of the SMILE procedure with micro-monovision in presbyopic patients with myopia and/or astigmatism. Monocular and binocular contrast sensitivity defocus curves (CSDC) were also measured. This new metric might help to identify possible patterns that could affect the results reported by patients undergoing combined myopia, astigmatism, and presbyopia correction techniques since it measures the entire defocus range and is more sensitive than visual acuity to the decrease in optical quality due to defocus secondary to accommodation reduction [10].

## 2. Materials and Methods

### 2.1. Subjects

Consecutive patients who decided to undergo SMILE with micro-monovision for the correction of myopia and/or astigmatism and presbyopia were invited to participate in this prospective observational pilot study. The nature and possible consequences of the study were explained to all participants, who provided written informed consent. The study was approved by the Ethics Committee of Research, Almería Center, Torrecardenas Hospital Complex and adhered to the tenets of the Declaration of Helsinki.

Seven patients were recruited from October 2021 to October 2022 at two centers in Spain: Tecnolaser Clinic Vision, Sevilla (Center A, 4 patients), and Qvision, Ophthalmology Department, VITHAS Almería (Center B, 3 patients). The inclusion criteria were patients between 40 and 55 years old, presbyopia with myopia and/or myopic astigmatism (≤6 D of myopia and ≤3 D of astigmatism), for whom micro-monovision with SMILE was programmed according to the standards of conventional clinical practice with a target in the non-dominant eye between −0.50 and −1.50 D, monocular visual acuity with best distance correction (CDVA) better or equal to 20/20, and sufficient availability, willingness, skills, and cognitive awareness to comply with follow-up/study procedures and study visits. Exclusion criteria were a history of ocular surgery (including laser refractive surgery), crystalline lens sclerosis according to the LOCS III classification system ≥CN1 or Pentacam Nucleus Staging (PNS) with Pentacam ≥ 2, intolerance to micro-monovision testing with contact lenses or trial frame for at least 30 min, failure of stereopsis test in near vision for all possible levels, pregnant woman at the time of surgery or follow-up, preoperative central corneal thickness less than 480 µm or a predicted postoperative residual stromal bed less than 250 µm, any ocular disorder that could potentially cause a loss of visual acuity or diplopia, topographic map compatible with subclinical keratoconus or other pathological alteration of the cornea, mu-chord >0.5 mm measured with Pentacam, use of systemic or ocular medications that may affect vision or accommodation in the last 6 months, and subjects participating in any clinical trial or research involving drugs or medical devices within 30 days prior to entry into this research and/or during the period of participation in this study.

### 2.2. Procedure

All patients underwent health exploration during the preoperative period for screening as candidates for micro-monovision with SMILE. This conventional exploration included slit-lamp, cycloplegic subjective refraction, corneal topography and biometry (Pentacam AXL, Oculus, Wetzlar, Germany), binocular and accommodative evaluation, photopic and mesopic pupil diameters (Keratograph 5M, Oculus, Wetzlar, Germany), and pupil diameter measured using a ruler under environmental light conditions. Ocular motor dominance was assessed by means of the pointing-a-finger test [11], then with the best distance correction in both eyes, a +1.50 D was placed over the non-dominant motor eye, reducing its value in −0.25 D steps up to achieving a tolerated value ≥ +0.50 D. This positive lens was tested for tolerance with either contact lens fitting to use at home or 30 min over the trial frame.

The study procedures included the measurement of monocular CDVA and binocular corrected visual acuities at 4 m (CDVA), 66 cm (CIVA), and 40 cm (CNVA) in the preoperative period [12], whereas monocular CDVA was measured at the 3-month safety evaluation and binocular uncorrected visual acuities at the same three distances in the 6-month follow-up for efficacy assessment (UDVA, UIVA, and UNVA). VAs were measured with an ETDRS iPad chart (VisionC, www.qvisionacademy.com, Almería, Spain) [13]. In the same way, stereopsis (StereoTAB,www.qvisionacademy.com, Almería, Spain) was evaluated at the preoperative period at far (3 m), intermediate (1.5 m), and near distances (50 cm) with best correction at each distance and in the 6-month without correction at the same distances [14]. The CSDC (MultifocalLA, www.qvisionacademy.com, Almería, Spain) was measured binocularly with the best distance correction in the preoperative period, whereas monocularly and binocularly CSDC without correction were evaluated at the 6-month follow-up. For measuring the CSDC, the patient was positioned at 4 m distance, then the MultifocalLA started with an alert message that indicated the defocus lens to be inserted (starting in +1.00 D). The experimenter pressed the orientation corresponding to the answer of the subject over a button bar of 4 possible crowded Sloan letters or a fifth button for pressing when the subject could not recognize the letter. Letters were presented randomly at a constant size of 0.3 logMAR (high spatial frequency) and increased or decreased contrast in 0.1 logCS steps depending on the patient’s answer. The final threshold was automatically determined by a staircase psychophysical procedure with five reversals. After testing the threshold for the first defocus lens, a new alert appeared over the screen with the defocus lens, which replaced the previous one (from +1.00 D to +0.50 D) and the five reversals were repeated but now starting at the CS threshold obtained with the previous defocus lens. This procedure was repeated for all the defocus lenses from +1.00 D to −4.00 D, in −0.50 D steps. A complete description of this procedure can be found in the validation study in which, instead of Sloan letters, Snellen letters were used [15].

All testing measurements were taken in both centers with an iPad set up at 85 cd/m^2^ of background luminance and an environmental light around 150 lux.

Patient-reported outcomes (PROs) were obtained in the preoperative stage and at 6 months by means of using the following questionnaires: the Convergence Insufficiency Symptoms Survey (CISS) to assess the near vision symptoms [16]; the Patient-Reported Spectacle Independence Questionnaire (PRSIQ) for the assessment of spectacle dependence [17]; the Vision and Night Driving Questionnaire (VND-Q) for assessing the difficulties driving at night [18]; and single questions to evaluate the dysphotopsia, and satisfaction with the procedure and desire to be submitted to the same procedure if patient had to take the decision again [19]. Adverse events were recorded during all the follow-up visits.

### 2.3. Surgery

Three experienced surgeons using the SMILE technique (JF, FAA, and JAC) performed the treatments with the VisuMax 500 kHz femtosecond laser system (Carl Zeiss Meditec AG) following the procedures of their habitual surgery practice. The final micro-monovision target in the non-dominant eye was selected depending on the tolerance test, surgeon’s recommendation, and the patient’s decision. Dominant eye was targeted to emmetropia and astigmatism was targeted for full correction in both eyes. The SMILE procedure involved three steps. The first step was the docking procedure, in which the center of the applanation zone was concentric with the margin of the cone and near the pupil center. In cases of astigmatism correction, a previous marking of the eye was conducted, and a slight rotation of the applanation cone was made to compensate for cyclotorsion, taking the horizontal lines seen through the microscope as a reference. After suction, the photodisruptive procedure creates a lenticule with a cap thickness between 120 and 130 µm and an optical zone diameter between 6.5 and 7.6 mm. Finally, the lenticule was extracted through an incision (2 mm wide) at the extreme end of the cap. All patients were treated preoperatively with topical anesthesia, with antibiotic and corticosteroid eye drops in the immediate postoperative period, and anti-inflammatory eye drops, decreasing the dosage progressively up to the third week after surgery.

### 2.4. Statistical Analysis

Descriptive statistics of the median (interquartile range) were selected to report the results after checking the normality using the Shapiro–Wilk test. Paired comparisons between preoperative and postoperative data were tested using the Wilcoxon signed-rank test. After closing the pilot study, a post hoc power calculation was conducted with a mean difference of 0.5 logCS and standard deviation of 0.24 logCS, obtaining a power of 0.97. SPSS software (version 26; SPSS Inc., Chicago, IL, USA) was used for the statistical analysis.

## 3. Results

One of the four recruited patients from center A withdrew from the study due to the inability to attend the 6-month visit. No adverse events were recorded for this patient at the last follow-up visit of 3 months and the achieved refraction was emmetropia in the dominant eye and −0.5 D in the non-dominant eye. The binocular UDVA, UIVA, and UNVA were −0.2, −0.2, and −0.1 logMAR, respectively, for this patient. Table 1 shows the demographics of the final sample of six subjects who completed all follow-up visits. The median predicted postoperative anisometropia was −0.81 D.

### 3.1. Safety

Only one adverse event was recorded during surgery. A small residual part of the lenticule (<1 mm) was extracted after the main lenticule was removed with no significant complications. No effects of visual acuity were observed in this eye (Table S1, Case A1). At the 3-month postoperative follow-up, a PRK procedure was conducted in the dominant eye of one patient due to undercorrection (Table S2, Case B1). The patient completed the 6-month visit and was included in the efficacy analysis. No eyes lost more than one line of CDVA at the 3-month visit compared with the preoperative period.

### 3.2. Efficacy

**Figure 1:**
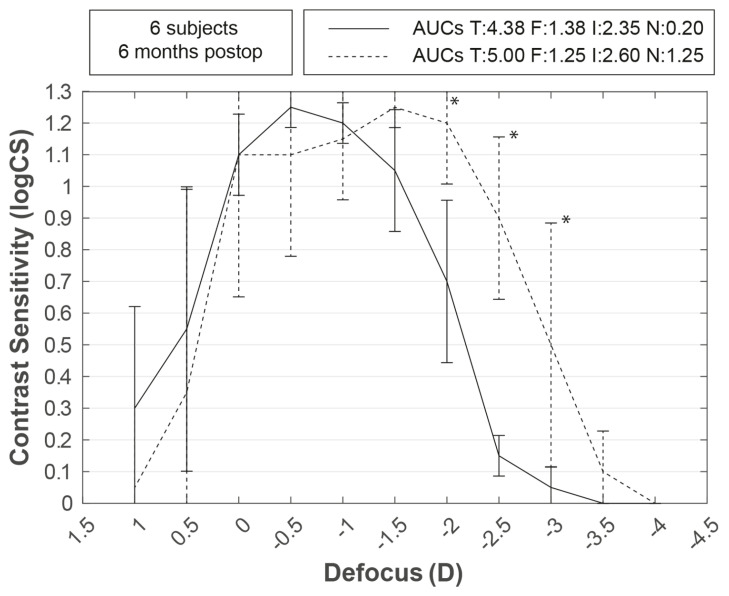
Preoperative best distance corrected versus postoperative uncorrected contrast sensitivity defocus curve. AUCs are the areas under the defocus curves above the 0.3 logCS value for the far (F), intermediate (I), and near (N) distance ranges. Lines represent median values, and vertical bars represent interquartile ranges. Asterisk (*) means *p* < 0.05.

No significant differences were found for median binocular uncorrected VAs at 6 months in comparison with the best-corrected VAs at the three measured distances in the preoperative visit (Table 2). All patients maintained stereopsis without correction (Tables S1 and S2) with no significant differences (Table 2). The uncorrected binocular CSDC showed a statistically significant increase in CS from −2 to −3 D of defocus in comparison to the preoperative binocular CSDC with the best distance correction (Table 2 and Figure 1). Individual CSDC of all patients are shown in Figure S1.

### 3.3. Patient-Reported Outcomes

The median CISS scores were close in the preoperative, 4 (26.75), and postoperative, 3 (12), visits (z = −1.089, *p* = 0.28). No significant changes (z = −0.95, *p* = 0.34) were found for the VND-Q in the preoperative, −3.1 (4.27), and postoperative, −3.94 (6.97), periods. All patients achieved postoperative spectacle independence at intermediate and near distances, and only the patient who underwent PRK required occasional spectacle correction for far distance. Four patients were satisfied or very satisfied with their uncorrected vision at all distances, one was very satisfied with the intermediate and near vision but neutral with far vision, and one was very satisfied at intermediate but neutral at far and slightly satisfied at near. All patients were slightly or not at all bothered by photic phenomena, except for two patients who answered very bothersome and moderately bothersome, but all the patients were likely or very likely to undergo the same procedure. The individual responses of all patients are shown in Tables S1 and S2.

## 4. Discussion

In this pilot study, the efficacy of micro-monovision with SMILE was evaluated and binocular summation using CSDC was reported for the first time. The efficacy of micro-monovision with SMILE has been published in previous studies [6,7,8]. Unfortunately, none of these studies reported standardized VA measured in logMAR with an ETDRS chart; therefore, the efficacy of our study cannot be easily compared with previous micro-monovision studies with SMILE reporting results using non-standard methods for reporting VA, such as Jaeger notation or reading charts. Difficulties with making comparisons have also been found even with other micro-monovision techniques such as Presbyond, mainly for the same limitation of using Jaeger notation in these studies [20,21,22,23]. In fact, this is an important limitation of the scarce evidence of presbyopia correction with laser refractive surgery techniques [24].

Being aware of the lack of uniform testing of VA, the median UDVA and UNVA achieved in our study were 0 and −0.05 logMAR, respectively. These values were close to those reported by Reinstein et al. for UDVA (−0.07 logMAR) and UNVA (0.05 logMAR) with Presbyond and an anisometropia of 1.5 D in older patients with a median age of 55 years old [22]. On the other hand, our results were better at distance in comparison to Kohnen et al. (0.09 logMAR) and comparable for near (−0.04 logMAR) [25]. This reduction in distance vision of Kohnen’s study is explained by a higher postoperative myopic spherical equivalent in the dominant eye, using the PresbyMAX with an anisometropia close to 0.5 D and older patients with a mean age of 53.4 years old [25]. Fu et al. also reported similar outcomes at near distance (0.01 logMAR) to Kohnen [26], but better distance results (−0.09 logMAR) by programming an anisometropia of 1.25 D in patients with a mean age of 47.4. According to these results, micro-monovision with SMILE appears to be a technique that might offer similar results to other platforms focused on presbyopia correction, but future randomized clinical trials are required to confirm this hypothesis.

An important finding of our study is related to the measurement of binocular uncorrected CSDC. An alternative to micro-monovision with SMILE can be the refractive lens exchange (RLE), which consists of replacing the clear crystalline lens by a multifocal intraocular lens [27]. Poorer results have been reported for RLE in young hyperopic patients (<40 years old) in comparison to our study for UDVA (0.01 logMAR), UIVA (0.2 logMAR), and UNVA (0.07 logMAR) [28]. On the other hand, binocular CSDC might result in reduced CS with multifocal IOLs [13], especially in the near and intermediate ranges, even though this has not been reported in young presbyopic patients as in our study. Considering the possible CS loss and the risks of retinal detachment in young myopic patients [27], micro-monovision of SMILE appears to be a more appropriate option, at least until the later fifties or early sixties when the retinal detachment risk decreases and the preoperative CS is lower due to crystalline lens sclerosis [29,30]. On the other hand, micro-monovision with SMILE in older patients could be questionable considering the onset of cataract development in the short term. In fact, even though the inclusion criteria was established at 55 years old in the protocol, the oldest patient in our sample was 51 years old, with a reasonable clear lens, and binocularly achieving 1 logCS in the preoperative stage at far distance, and 0.9 logCS at 0 D and −2.5 D at the 6-month visit, which means a better CS at near in comparison to that achieved with MIOLs [13]. Thus, preoperative screening considering the cut-off criteria for CS reestablishment with MIOLs is of great importance when taking the decision for laser vision correction with micro-monovision or refractive lens exchange [31]. The latter, with the consensus of the patient after explanation of the advantages and drawbacks of each procedure, will determine the best procedure for each patient. In addition, it should be noticed that patients satisfied with micro-monovision with laser vision correction could be programmed in the future for the same micro-monovision with conventional or enhanced monofocals and extended depth of focus intraocular lens, or even for multifocal intraocular lenses targeted to emmetropia [32]. Moreover, special formulas for post-laser refractive surgery such as those included in the ASCRS online calculator (https://iolcalc.ascrs.org/ accessed on 16 March 2023), thick-lens or ray-tracing formulas should be used [33,34]. Presbyopic phakic IOLs could also be an alternative for those patients, but CSDC studies are still required to determine if CS is also maintained along the whole defocus range [35,36,37].

An argument against monovision has been the possible loss of stereopsis [38], but previous studies with Presbyond reported minimal changes [39]. Our study is in agreement with the lack of clinically relevant differences in stereopsis loss but also provides, for the first time, evidence of intermediate and far distance stereopsis. This apparently unaltered stereopsis at all distances can be explained by programming a low anisometropia below 1.5 D [38]. Psychophysical studies have also suggested that disparity between eye images can affect reflexive eye movements, but this topic has still not been studied in clinical practice with patients operated on with laser refractive surgery and micro-monovision [40,41]. In addition, the lack of differences in symptoms evaluated with the CISS questionnaire and the VND-Q supports that micro-monovision with SMILE might not induce symptoms and difficulties in important tasks related to near work and driving at night. This, together with the low dysphotopsia incidence, explains the satisfaction rates in our study, with all patients having better satisfaction answers in comparison to the preoperative period for far and intermediate distances, and only two patients who chose one level less in the postoperative answer for near vision, with the remaining four patients selecting a better or maintained answer. Despite the reduced answers to some isolated questions, all patients agreed with the probability of being submitted again to the same procedure with a likely or very likely answer.

The main limitations of our study were its small sample size and the short-term follow-up, which limit the generalizability of the findings. However, it is important to note that the difference achieved in our study was 0.5 logCS with a micro-monovision programmed median anisometropia of 0.81 D. For this difference, and the standard deviation of 0.24 logCS obtained in this study, the power was above 0.8 for an alpha value of 0.05. Therefore, a sufficient sample size was used to avoid a Type II error at −2 D. In summary, our sample size was enough to evidence the increase in the CS at the defocus level of −2 D but was not enough to avoid a Type II error in differences below 0.4 logCS, for instance, to confirm the decrease of 0.15 logCS at −0.5 D or the increase at −1.5 D.

## 5. Conclusions

This was a pilot study for future estimations of the sample size based on the collected results around the main endpoint. The programmed median anisometropia of 0.8 D resulted in the effectiveness of the procedure for patients of a median age of 45 years old. The results were comparable to other presbyopia laser correction techniques that also implement micro-monovision even though this comparison should be interpreted with caution due to the differences in testing methods and age of the population. The most interesting finding was that the micro-monovision binocular summation resulted in superior contrast sensitivity as compared to the multifocal refractive lens exchange performed in a similar patient group; the stereopsis is in the normal range with the induction of this anisometropia without increasing symptoms or difficulties with near vision tasks or driving at night. On the other hand, all the findings of this pilot study should be confirmed in future studies with larger sample sizes.

## Figures and Tables

**Table 1:** Demographic characteristics of the sample.

Variable	Median	Interquartile Range
Age	45	5
Sphere (D)	−2.88	2
Cylinder (D)	−0.5	0.75
SE (D)	−3.13	1.72
Dominant Predicted SE (D)	−0.13	0.56
Non-Dominant Predicted SE (D)	−0.94	0.63
Photopic pupil diameter (mm)	3.8	1.12
Mesopic pupil diameter (mm)	6.05	0.94
Ruler pupil diameter (mm)	4	0.5
Axial Length (mm)	24.60	1.27
Mean Keratometry (D)	43.25	0.89

SE: spherical equivalent.

**Table 2:** Binocular efficacy results of median (interquartile range). Preoperative with best correction at each distance and postoperative without distance correction.

Variable	Preoperative	6-Month	z, *p*-Value
Visual Acuity (logMAR)			
Far (4 m)	−0.1 (0.2)	0 (0.18)	0.7, 0.48
Intermediate (66 cm)	0 (0.02)	−0.1 (0.15)	−1.63, 0.1
Near (40 cm)	−0.1 (0.2)	−0.05 (0.18)	1.12, 0.66
Stereopsis (arcsec)			
Far (4 m)	79 (178)	119 (20)	0.32, 0.75
Intermediate (66 cm)	79 (79)	40 (9.75)	−1.63, 0.10
Near (40 cm)	79 (49)	59 (39)	−1.00, 0.32
Contrast Sensitivity (logCS)			
Defocus −4.0	0 (0.03)	0 (0.1)	1, 0.32
Defocus −3.5	0 (0.03)	0.1 (0.38)	1.63, 0.1
Defocus −3.0	0.05 (0.1)	0.5 (0.68)	2.02, 0.04
Defocus −2.5	0.15 (0.2)	0.9 (0.58)	2.04, 0.04
Defocus −2.0	0.7 (0.4)	1.2 (0.40)	2.03, 0.04
Defocus −1.5	1.05 (0.3)	1.25 (0.18)	1.60, 0.1
Defocus −1.0	1.2 (0.12)	1.15 (0.30)	0, 1
Defocus −0.5	1.25 (0.20)	1.10 (0.50)	−1.60, 0.1
Defocus 0.0	1.1 (0.38)	1.10 (0.75)	−1, 0.32
Defocus 0.5	0.55 (0.80)	0.35 (1.02)	2.22, 0.03
Defocus 1.0	0.30 (0.55)	0.05 (0.48)	−1.83, 0.07

## Data Availability

Not applicable.

## Supplementary Materials

The following supporting information can be downloaded at: https://www.mdpi.com/article/10.3390/life13030838/s1, Figure S1: Contrast sensitivity defocus curves for each one of the patients at the preoperative visit with best distance correction (PreBE) and the postoperative 6-month visit without correction for right eye (PosRE), left eye (PosLE), and both eyes (PosBE); Table S1: Preoperative and postoperative results for the patients from Center A; Table S2: Preoperative and postoperative results for the patients from Center B.
