# Supplementary material for: Effectiveness of SMILE Combined with Micro-Monovision in Presbyopic Patients: A Pilot Study
Source: Life (Basel). 2023 Mar 20;13(3):838. doi: 10.3390/life13030838 (PMC10051050; doi:10.3390/life13030838)
Supplement: Supplementary file 1 [file life-13-00838-s001.zip › Table S1.pdf]

Table S1. Preoperative and postoperative results for the patients from Center A.

	Case A1 (Micro 0.5 D) (Women / 45 years old)		Case A2 (Micro 0.5 D) (Men / 42 years old)		Case A3 (Micro 1.0 D) (Men / 45 years old)	
	**Preop.**	**6-month**	**Preop.**	**6-month**	**Preop.**	**6-month**
**Right Eye Sphere (D)**	-2,50	-0,25	-3,25	0	-3,50	-1
**Astigmatism (D)**	(-0,25)40	(-0,25)10	(-0,50)90		(-0,50)55	
**Left Eye Sphere (D)**	-2,50	-0,25	-3,50	-0,50	-3,50	0
**Astigmatism (D)**	(-0,50)140	(-0,25)135	(-0,75)85		(-0,50)160	
**Right Eye SE (D)**	-2,625	-0,375	-3,5	0	-3,75	-1
**Left Eye SE (D)**	-2,75	-0,375	-3,875	-0,50	-3,75	0
**Binocular Efficacy**						
**Far VA (logMAR)**	0	-0.1	-0,2	-0,3	-0,2	0,1
**Interm. VA (logMAR)**	0	-0.1	0	-0,3	0	0
**Near VA (logMAR)**	0	0	-0,2	-0,3	-0,2	0,1
**Stereopsis**						
**Far (arcsec)**	397	159	79	119	40	79
**Intermediate (arcsec)**	119	40	119	40	40	40
**Near (arcsec)**	119	40	79	79	40	40
**CISS Score**	36	24	29	10	5	4
**Driving Score (logit score)**	-0.41	-1.20	-3.10	0.59	-4.87	-7.72
**Single questions**						
**Total Spectacle Ind.**						
**Far**	No	Yes	No	Yes	No	Yes
**Intermediate**	Yes	Yes	No	Yes	No	Yes
**Near**	Yes	Yes	Yes	Yes	Yes	Yes
**Satisfaction**						
**Far**	Not at all	Satisfied	Not at all	Neutral	Not at all	Very
**Intermediate**	Slightly	Satisfied	Not at all	Very	Not at all	Very
**Near**	Very	Satisfied	Neutral	Slightly	Very	Very
**Dysphotopsia (Bothersome)**	Slightly	Very	Moderately	Moderately	Not at all	Not at all
**Submited again (Likely)**	-	Likely	-	Likely	-	Very
