# Supplementary material for: Effectiveness of SMILE Combined with Micro-Monovision in Presbyopic Patients: A Pilot Study
Source: Life (Basel). 2023 Mar 20;13(3):838. doi: 10.3390/life13030838 (PMC10051050; doi:10.3390/life13030838)
Supplement: Supplementary file 1 [file life-13-00838-s001.zip › Table S2.pdf]

Table S2. Preoperative and postoperative results for the patients from Center B.

	Case B.1 (Micro 1.0 D) (Women/51 years old)		Case B.2 (Micro 0.75 D) (Men/48 years old)		Case B.3 (Micro 1.0 D) (Men / 42 years old)	
	**Preop.**	**6-month**	**Preop.**	**6-month**	**Preop.**	**6-month**
**Right Eye Sphere (D)**	-3,75	-1,25	-1	0	-1,5	-0.25
**Astigmatism (D)**	(-1,50)170	(-0,50)180	(-0,50)180	(-0,50)180	(-1,25)90	0
**Left Eye Sphere (D)**	-3,75	-0,25	-1	-0,75	-1,50	-1.25
**Astigmatism (D)**	(-1,50)180	(-0,50)10	(-0,50)120	(-0,50)120	(-1,25)75	0
**Right Eye SE (D)**	-2,625	-1,5	-0,75	-0,25	-1,375	-0.25
**Left Eye SE (D)**	-2,625	-0,5	-0,75	-1	-1,375	-1.25
**Binocular Efficacy**						
**Far VA (logMAR)**	0	0	-0,1	0	-0,1	0
**Interm. VA (logMAR)**	0	0	-0,1	-0.1	0	-0.1
**Near VA (logMAR)**	0	0	-0,1	-0.1	-0,1	-0.1
**Stereopsis**						
**Far (arcsec)**	159	119	40	119	79	119
**Intermediate (arcsec)**	79	79	40	40	40	40
**Near (arcsec)**	79	79	79	79	40	40
**CISS Score**	0	2	0	0	3	2
**Driving Score (logit score)**	-4.87	-7.72	-3.10	-2.40	-0.67	-5.49
**Single questions**						
**Total Spectacle Ind.**						
**Far**	Yes	Yes	Yes	No	No	Yes
**Intermediate**	Yes	Yes	No	Yes	No	Yes
**Near**	No	Yes	No	Yes	Yes	Yes
**Satisfaction**						
**Far**	Not at all	Very	Not at all	Neutral	Not at all	Satisfied
**Intermediate**	Not at all	Very	Slightly	Very	Not at all	Very
**Near**	Satisfied	Very	Satisfied	Very	Satisfied	Satisfied
**Dysphotopsia (Bothersome)**	Not at all	Slightly	Slightly	Slightly	Very	Not at all
**Submited again (Likely)**	-	Very	-	Likely	-	Very
