# Supplementary figures and images for: Effectiveness of SMILE Combined with Micro-Monovision in Presbyopic Patients: A Pilot Study
Source: Life (Basel). 2023 Mar 20;13(3):838. doi: 10.3390/life13030838 (PMC10051050; doi:10.3390/life13030838)

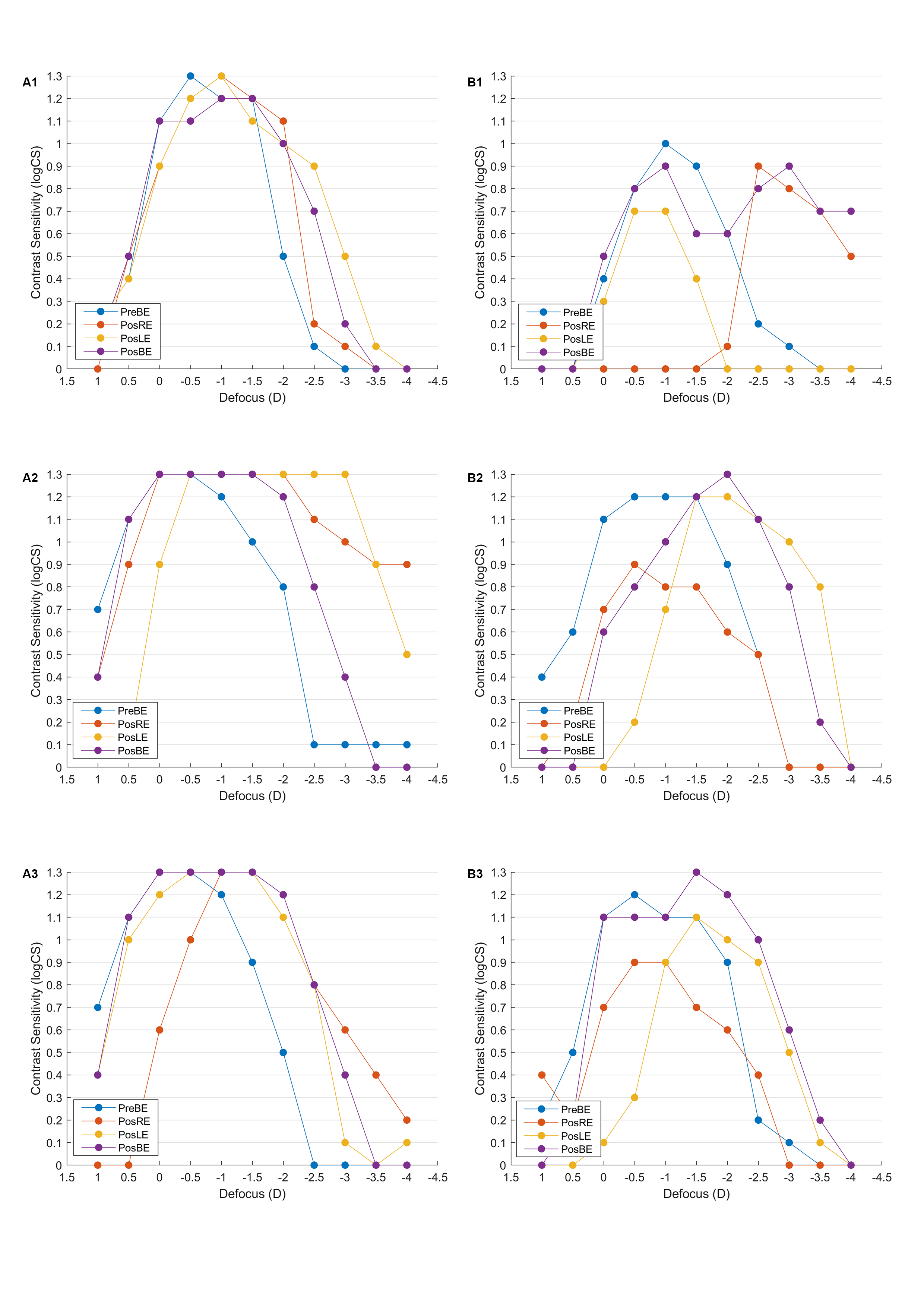

Supplement: Supplementary file 1 [file life-13-00838-s001.zip › Figure S1.tif]
